# The antibiotic chloramphenicol may be an effective new agent for inhibiting the growth of multiple myeloma

**DOI:** 10.18632/oncotarget.10623

**Published:** 2016-07-16

**Authors:** Faqing Tian, Chunyan Wang, Meiqin Tang, Juheng Li, Xiaohui Cheng, Sihan Zhang, Delan Ji, Yingcai Huang, Huiqing Li

**Affiliations:** ^1^ Department of Hematology, Longgang District People's Hospital of Shenzhen, Guangdong, China; ^2^ Department of Rheumatology, Longgang District People's Hospital of Shenzhen, Guangdong, China; ^3^ Department of Rheumatology, Lanzhou University Second Hospital, Gansu, China

**Keywords:** chloramphenicol, multiple myeloma, adenosine triphosphate, apoptosis

## Abstract

Chloramphenicol is an old antibiotic that also inhibits mammalian mitochondrial protein synthesis. Our studies demonstrated that chloramphenicol is highly cytotoxic to myeloma cells, acting in a dose- and time-dependent manner. Chloramphenicol sharply suppressed ATP levels in myeloma cells at concentrations ≥ 25 μg/mL. Colorimetric and clonogenic assays indicate that chloramphenicol inhibits growth of myeloma cell lines at concentrations ≥ 50 μg/mL, and inhibits primary myeloma cell growth at concentrations ≥ 25 μg/mL. Flow cytometry and Western blotting showed that chloramphenicol induces myeloma cell apoptosis at concentrations ≥ 50 μg/mL. Chloramphenicol increased levels of cytochrome c, cleaved caspase-9 and cleaved caspase-3, suggesting that myeloma cell apoptosis occurs through the mitochondria-mediated apoptosis pathway. It thus appears chloramphenicol is not only an old antibiotic, it is also a potential cytotoxic agent effective against myeloma cells. This suggests chloramphenicol may be an effective “new” drug for the treatment of myeloma.

## INTRODUCTION

Multiple myeloma (MM) is a B-cell tumor characterized by clonal expansion of malignant plasma cells within the bone marrow [1, 2]. Although the recent use of proteasome inhibitors and immunomodulatory drugs has improved response rates and overall survival, this tumor remains incurable for the vast majority of patients, so new treatments are urgently needed [3, 4].

Cellular metabolism is the most important characteristic of living cells. Both normal and tumor cells must produce enough energy to maintain life and support cell proliferation by diverting enough metabolic intermediates to biosynthetic pathways [5–7]. Cellular energy is mainly stored in the form of adenosine triphosphate (ATP), which is produce through anaerobic and aerobic glycolysis–two ATPs are generated per molecule of glucose via glycolysis in the cytoplasm and up to 36 ATPs per glucose are produced through complete catabolism via the TCA cycle and OXPHOS in mitochondria) [7]. Perhaps targeting tumor cell metabolism to suppress ATP production could be an effective future therapy for MM [8].

Prior reports showed that chloramphenicol inhibits mammalian mitochondrial protein synthesis and causes mitochondrial stress, leading to decreased ATP biosynthesis [9–14]. The use of chloramphenicol as an antimicrobial agent to treat bacterial infections has decreased over the years because it suppresses bone marrow function in humans secondary to inhibition of mitochondrial protein synthesis [15, 16]. However, it has been suggested that this adverse effect could be used to benefit leukemia patients [16–18].

Previous studies have suggested that chloramphenicol treatment causes mitochondrial stress, but prevents cancer cell apoptosis, and enhances cancer invasion, particularly in some solid tumors containing anoxic environments that rely on anaerobic glycolysis to generate ATP [10–12]. On the other hand, bone marrow is regarded as an oxygen-rich microenvironment, in which myeloma cells produce large amounts of energy to support cell proliferation and contribute to the synthesis and excretion of monoclonal immunoglobulin. In our pre-assays, data showed that chloramphenicol inhibited the proliferation of myeloma cells, but the underlying mechanism is not yet fully understood. We hypothesized that chloramphenicol may decrease cellular energy metabolism, thereby inhibiting MM cell proliferation and colony formation.

## RESULTS

### Cell growth inhibition

**Figure 1:**
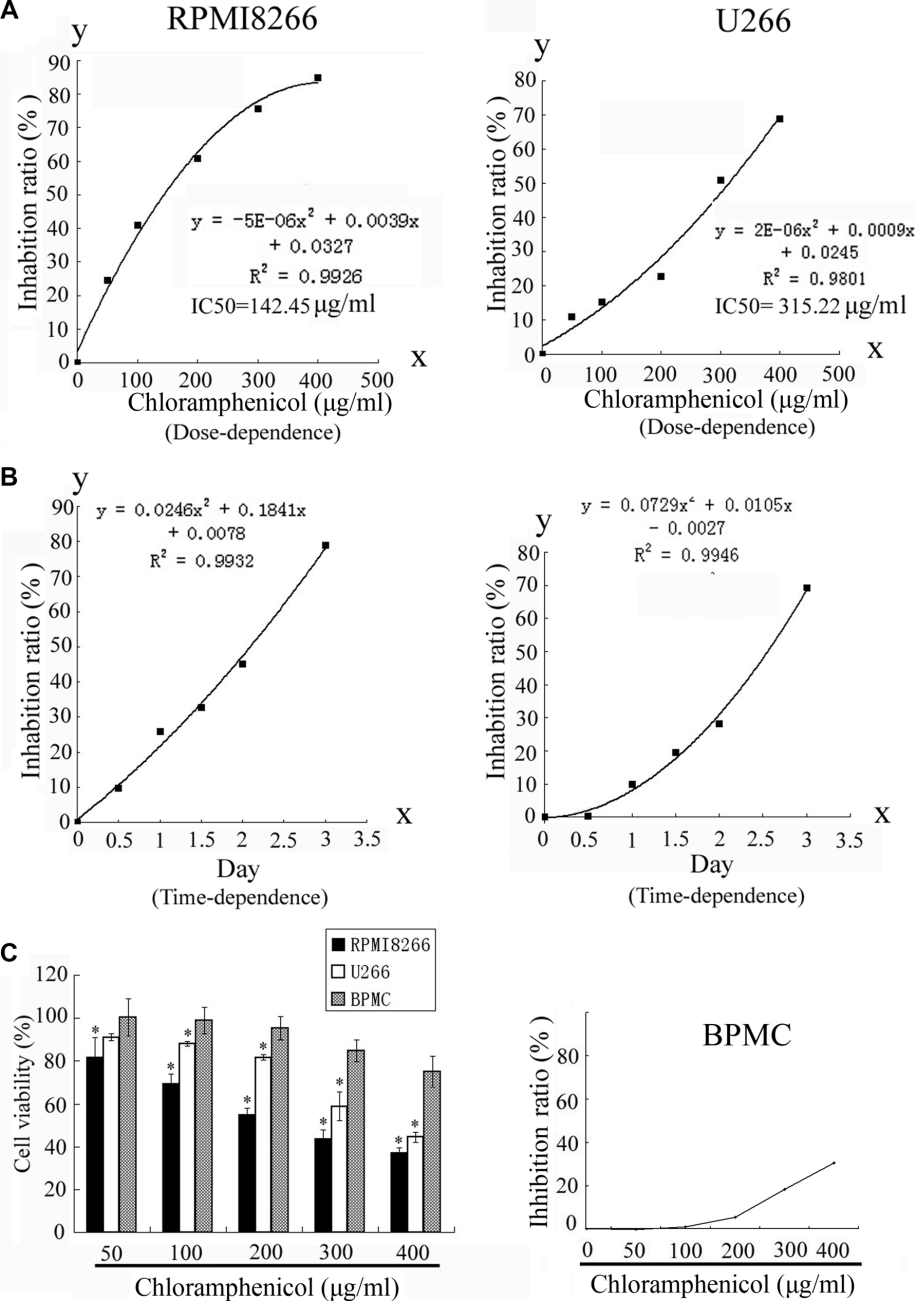
Inhibition of RPMI8266 and U266 human MM cell proliferation (**A**) Proliferation of RPMI8266 and U266 cells human MM cells was dose-dependently decreased by treatment with chloramphenicol for 48 h. IC50 values were 142.45 μg/ml and 315.22 μg/mL at 48 h with RPMI8266 and U266 cells, respectively. (**B**) The proliferation of human MM cell lines was decreased by chloramphenicol in a time-dependent manner (chloramphenicol: 100 μg/mL). (**C**) Proliferation of normal PBMCs was weakly decreased by chloramphenicol. Results are expressed as the mean ± SEM (error bars) for four separate experiments; *P < 0.05.

**Figure 2:**
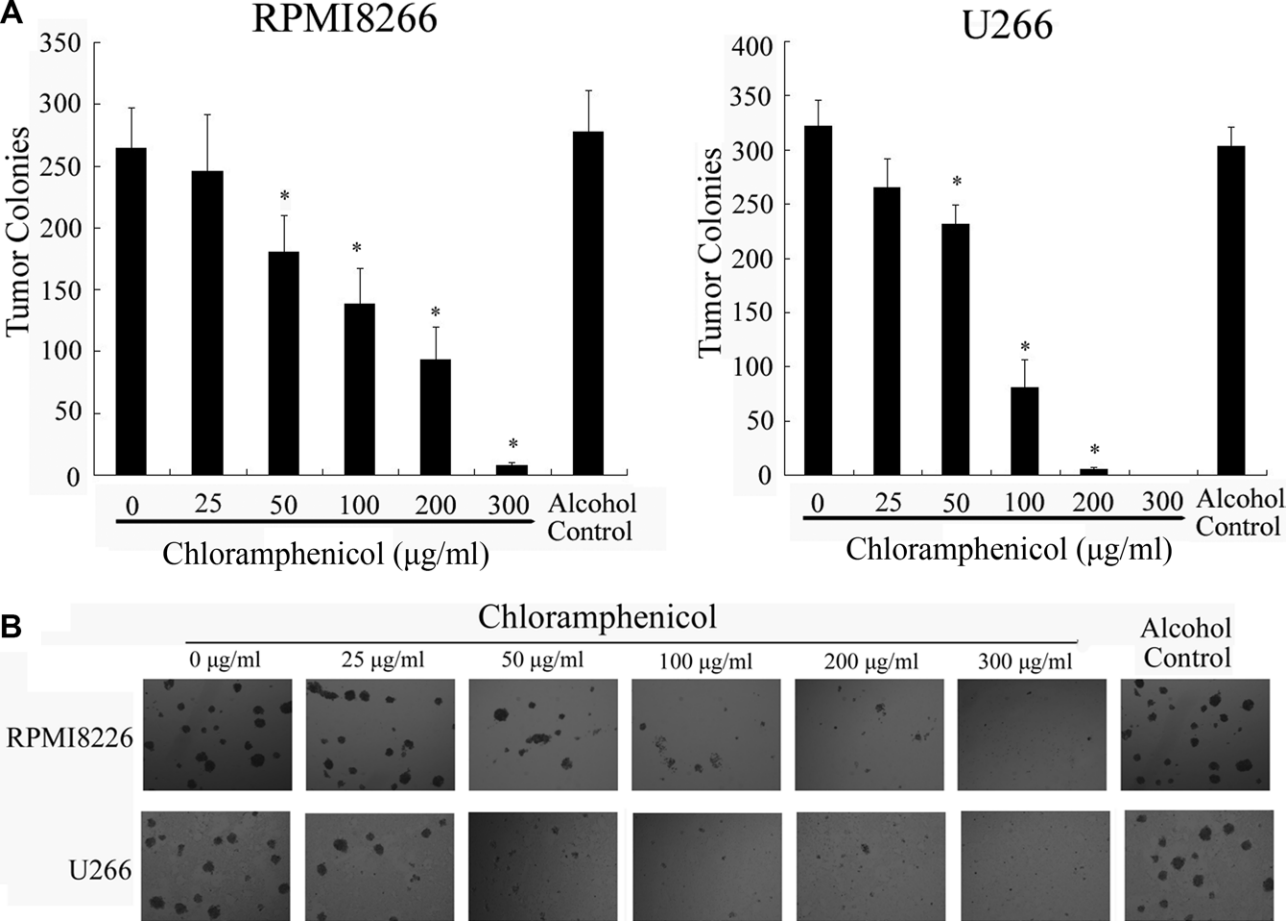
Suppression of colony formation by human MM cell lines (**A**) Colony formation was dose-dependently inhibited by chloramphenicol. The results are expressed as the mean ± SEM (error bars) for three separate experiments; *P < 0.05. (**B**) Photomicrographs show the appearance of colonies at a low power (tumor cells: 10,000/well).

To determine the effect of chloramphenicol on cell growth, RPMI8266 and U266 cells, as well as unstimulated freshly isolated peripheral blood mononuclear cells (PBMCs), taken as a normal cell counterpart, were exposed to chloramphenicol or its vehicle, and the number of viable cells was measured using colorimetric assays. Dose- and time-response curves were obtained over a range of doses and days. The colorimetric assays indicated that chloramphenicol inhibited MM cell proliferation in a dose- and time-dependent manner (Figure 1A–1B), but only weakly inhibited the proliferation of normal PBMCs (Figure 1C). We suggest that the proliferation and energy metabolism of MM cells are probably at a higher level than in normal PBMCs. Chloramphenicol was highly cytotoxic at ≥ 50 μg/mL. The half maximal inhibitory concentration (IC50) values were 142.45 μg/mL and 315.22 μg/mL for RPMI8266 and U266, respectively. Importantly, clonogenic assays showed that chloramphenicol dose-dependently suppressed tumor cell colony formation at concentrations ≥ 50 μg/mL (Figure 2A–2B). These assays indicate that chloramphenicol suppresses MM cell growth. Alcohol had no impact on cell proliferation at low concentrations (< 5 μL/mL).

### Cellular ATP levels and *in vitro* tumor cell invasion

**Figure 3:**
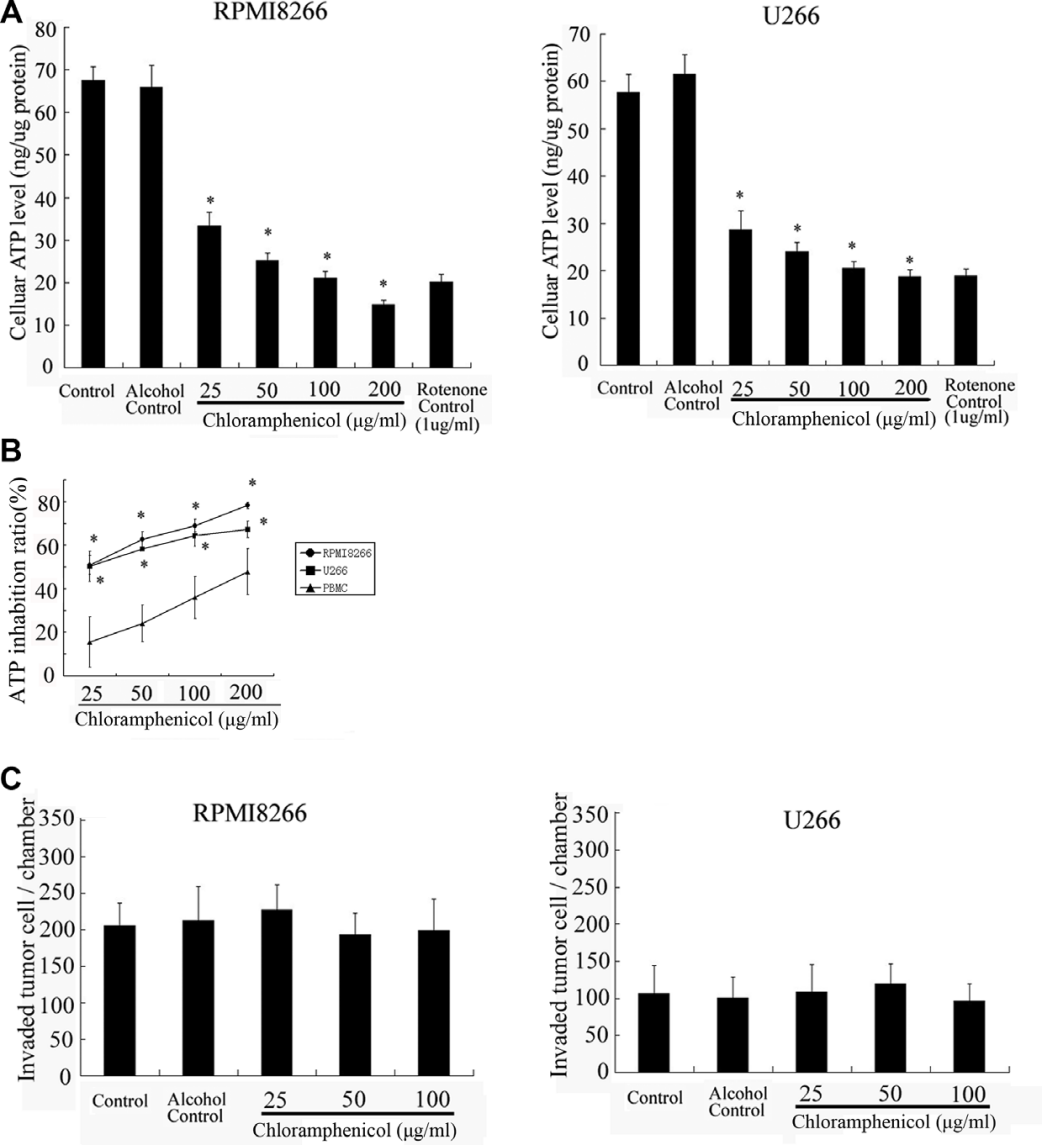
Cellular ATP levels and *in vitro* tumor cell invasion (**A**) ATP levels in tumor cells were sharply suppressed by chloramphenicol in a dose-dependent manner. (**B**) Chloramphenicol-induced ATP inhibition was much weaker in normal PBMCs than MM cells. The results are expressed as the mean ± SEM (error bars) for four separate experiments; *P < 0.05. (**C**) Transwell *in vitro* invasion assays indicate that chloramphenicol had almost no effect on the invasiveness of MM cells after 48 h of treatment (2.5 × 10^4^ tumor cells per well). The results are expressed as the mean ± SEM (error bars) for four separate experiments; * *P* < 0.05.

To test whether chloramphenicol impacts mitochondrial energy metabolism in MM cells, tumor cells were cultured with different concentrations of chloramphenicol prior to measuring cellular ATP content. The measurements confirmed that ATP levels in the tumor cells decreased in the presence of chloramphenicol, and the effect was dose-dependent (Figure 3A). A similar effect was elicited by rotenone, an inhibitor of the mitochondrial complex I electron transport chain, which served as a positive control. As compared with MM cells, ATP levels in normal PBMCs were only weakly decreased by chloramphenicol (Figure 3B). In addition, transwell *in vitro* invasion assays indicated that chloramphenicol had almost no impact on the invasiveness of MM cells (Figure 3C).

### Tumor cell apoptosis

**Figure 4:**
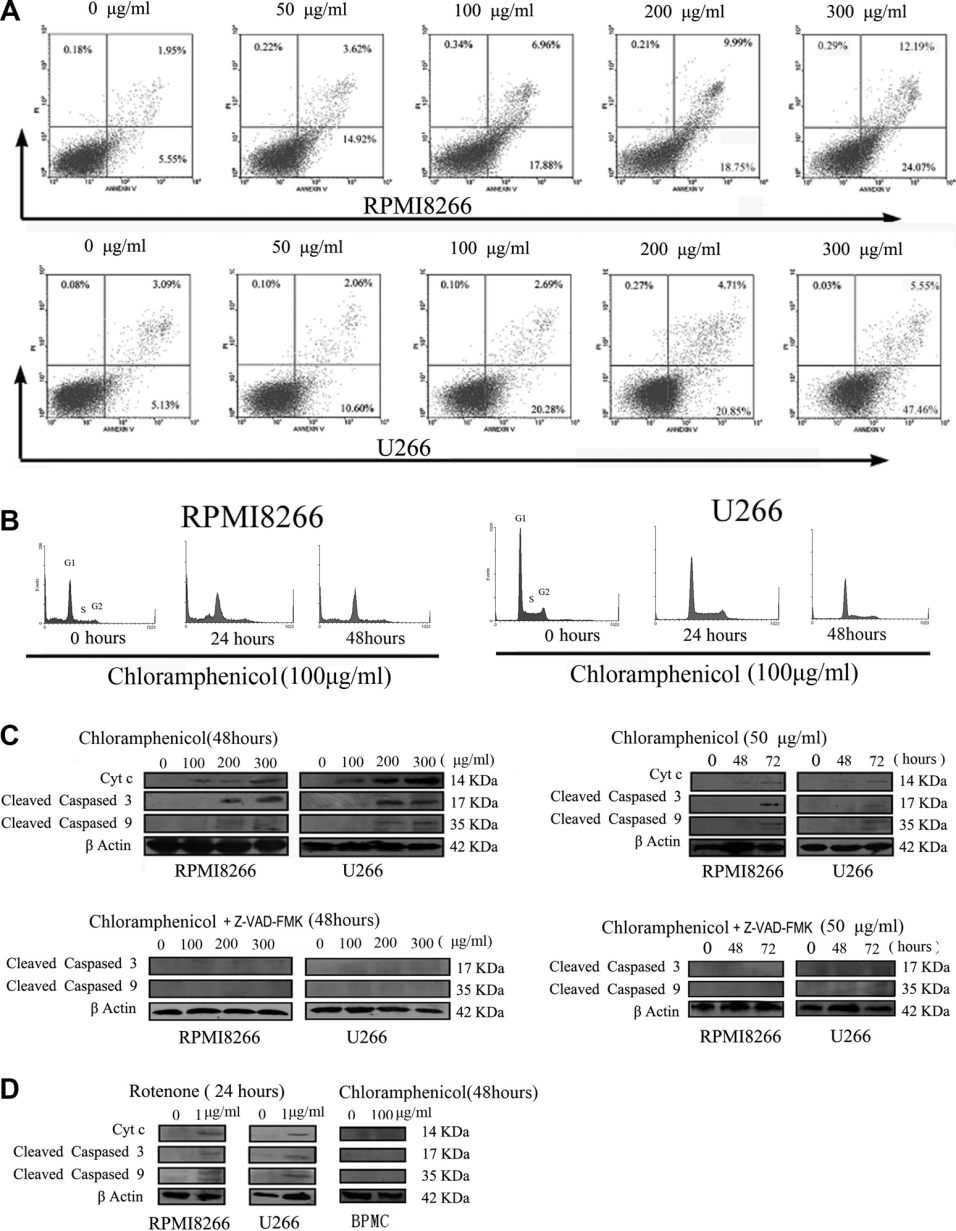
Chloramphenicol-induced apoptosis (**A**) Flow cytometric analysis showed that chloramphenicol dose-dependently increased early (annexin V positive and PI negative cells) and late (annexin V and PI positive cells) apoptosis rates. (**B**) Flow cytometric analysis of the MM cell cycle during treatment with 100 μg/mL chloramphenicol for 0, 24 and 48 h. The differences were not statistically significant in four separate experiments, *P* > 0.05. (**C**) Western blot analysis showing that prolonged treatment with chloramphenicol (≥ 100 μg/mL or 50 μg/mL) increased levels of Cytc, cleaved caspase 9, and cleaved caspase 3 in MM cells (upper). The caspase activation was completely inhibited by 25 μM Z-VAD-FMK (lower). β actin served as a loading control. (**D**) Like chloramphenicol, rotenone increased levels of Cytc, cleaved caspase 9, and cleaved caspase 3 in MM cells (left and middle). Chloramphenicol (100 μg/mL for 48 h) did not increase Cytc, cleaved caspase 9 or cleaved caspase 3 in PBMCs (right).

We next determined whether chloramphenicol induces apoptosis of MM cells. As indicated in Figure 4A–4B, chloramphenicol dose-dependently increased the rates of both early (annexin V positive and PI negative cells) and late (annexin V and PI positive cells) apoptosis, with a significant effect observed at concentrations ≥ 50 μg/mL. Cleaved caspases 3 and 9 are the activated forms of these proteolytic enzymes, which are biomarkers of apoptosis. Western blot analysis suggested that chloramphenicol (≥ 50 μg/mL) increased the abundance of Cytc, cleaved caspase 9, and cleaved caspase 3 in tumor cells, and that this effect on the caspases was blocked by 25 μM Z-VAD-FMK, a nonspecific caspase inhibitor (Figure 4C). As a possible control for chloramphenicol, rotenone induced increases in the abundance of Cytc, cleaved caspase 9 and cleaved caspase 3 in tumor cells. As a control for MM cells, PBMCs showed no increases in Cytc, cleaved caspase 9 or cleaved caspase 3 after 48 h of treatment with chloramphenicol (100 μg/mL) (Figure 4D)

### Proliferation and clonogenic assays with primary tumor cells

**Figure 5:**
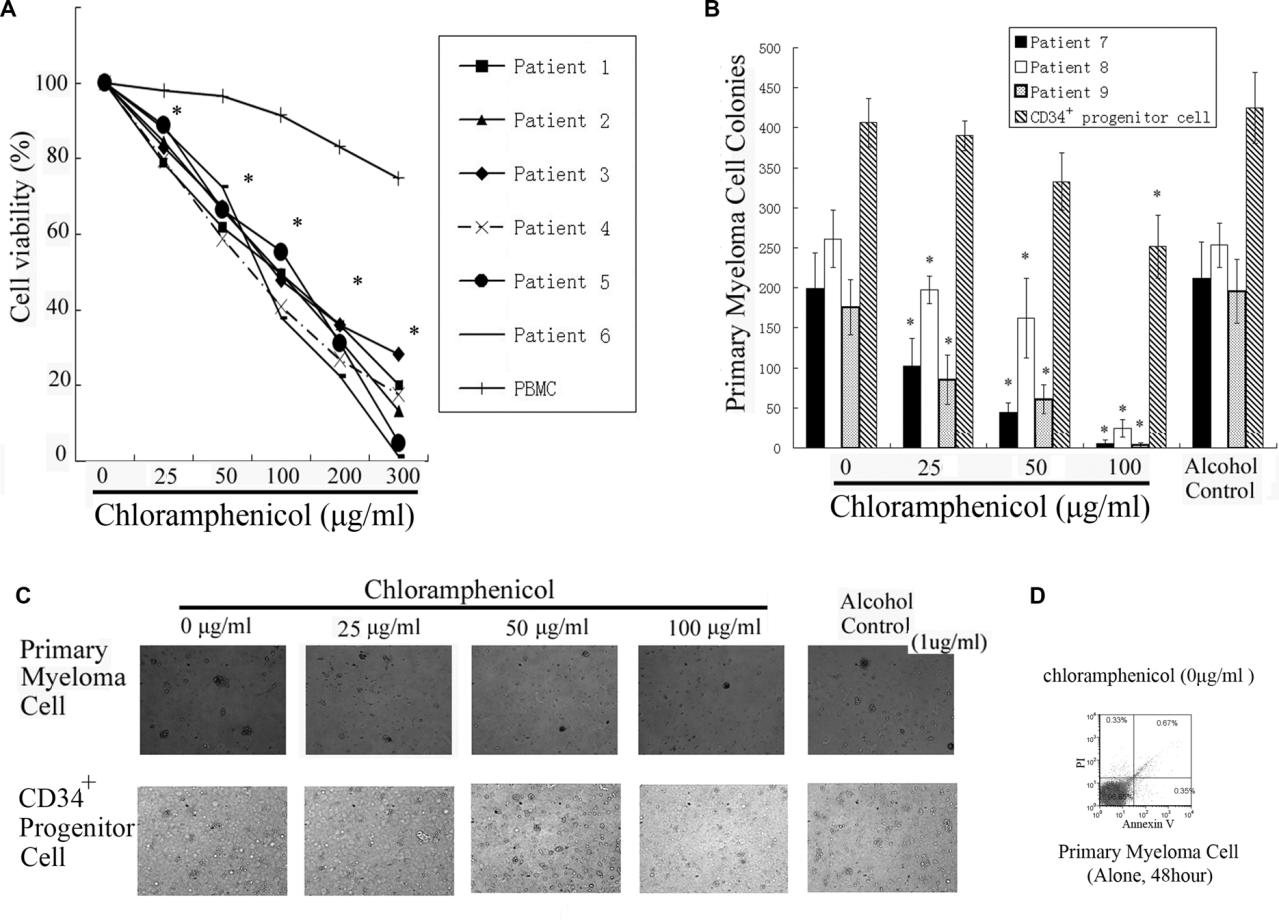
Inhibition of primary MM cell growth (**A**) Colorimetric assays showed that chloramphenicol dose-dependently suppressed tumor cells proliferation. The results are expressed as the mean ± SEM (error bars) for three separate experiments; *P < 0.05. (**B**) Clonogenicity was markedly inhibited with chloramphenicol. As a control for MM cells, the clonogenicity of CD34^+^ progenitor cells was weakly repressed by chloramphenicol. (**C**) Photomicrographs showing the appearance of primary tumor cell and CD34^+^ progenitor cell colonies at a low power. (**D**) Primary myeloma cells cultured alone for 48 h were stained with propidium iodide (PI) and annexin-V-FITC. Flow cytometric analysis showed that there was almost no apoptosis among MM cells.

To gain insight into the effect of chloramphenicol on primary MM cells, bone marrow samples from patients with MM were examined. Colorimetric and clonogenic assays showed that chloramphenicol dose-dependently decreased both the proliferation and clonogenicity of bone marrow MM cells. The curves and figures indicate that chloramphenicol at concentrations ≥ 25 μg/mL markedly inhibited the growth of primary MM cells (Figure 5A–5C). Flow cytometry showed that there was almost no apoptosis among primary MM cells cultured alone for 48 h (Figure 5D).

## DISCUSSION

Chloramphenicol reversibly binds to the 50S subunit of the 70S ribosome in prokaryotes, thereby inhibiting peptidyl transferase and in turn protein synthesis [13], [19]. As the structure of mammalian mitochondria is similar to prokaryotes [13, 14, 20], mitochondrial protein synthesis can also be inhibited by chloramphenicol. Our results indicate that chloramphenicol sharply suppresses ATP levels in human MM cell lines and primary MM cells at concentrations ≥ 25 μg/mL and significantly inhibits tumor growth at concentrations ≥ 50 μg/mL. Flow cytometry and Western blotting showed that chloramphenicol also induced MM cell apoptosis at ≥ 50 μg/mL. These data are consistent with earlier clinical reports indicating that chloramphenicol caused bone marrow suppression and aplastic anemia in a dose- and time-dependent manner [9, 21–25]. It has been suggested that the bone marrow toxicity of chloramphenicol may be useful for treatment of leukemia [16–18]. Consistent with that idea, our experiments indicate that chloramphenicol may be beneficial for patients with MM.

We found that low doses of chloramphenicol (e.g., 25 μg/mL) had almost no effect on the number or size of tumor cell colonies during the 2–3 weeks of treatment in MM cell clonogenic assays, but cellular ATP levels were effectively suppressed at that concentration. This inhibition of energy metabolism would change tumor biology, making it unconducive to tumor cell growth [8]. In contrast to previous reports [10, 11], a small increase in the chloramphenicol dose (to ≥ 50 μg/mL) greatly suppressed tumor growth while further decreasing ATP levels. These phenomena suggest that a deep deficiency in ATP can effectively suppress tumor cell proliferation. Perhaps one of the mechanisms is the lack of one or more key energy metabolism intermediates resulting from the inhibition of the TCA cycle and mitochondrial protein synthesis. However, our studies focused on the mitochondria-mediated apoptosis pathway in which cytochrome c is released from mitochondria and activates caspase 9 downstream [26–29]. Western blot analysis showed that higher doses of chloramphenicol (≥ 100 μg/mL) induced production of cytochrome c, cleaved caspase 9, and cleaved caspase 3, as did prolonged treatment with 50 μg/mL chloramphenicol. These data suggest that chloramphenicol not only reduces ATP levels in MM cells, it also induces mitochondria-mediated apoptosis. In short, both decreased ATP levels and MM cell apoptosis likely account for the inhibition of tumor growth induced by chloramphenicol. However, our findings do not exclude other apoptosis pathways induced by chloramphenicol.

Chloramphenicol is highly lipid soluble [11, 30]. Its target serum concentration for treating infectious diseases is 10–30 μg/mL in serum, which corresponds to a dose of 50–100 mg/kg/day [19]. Our experiments confirmed that lower concentrations of chloramphenicol (25 μg/mL) significantly inhibit primary MM cell proliferation, which is consistent with a previous report of inhibition of mouse myeloma cell proliferation by chloramphenicol [31]. Because chloramphenicol is highly fat soluble [11], it likely reaches higher concentrations in the bone marrow, where MM cells mainly survive and accumulate, than in the serum [32]. Thus, higher local drug concentrations may inhibit the growth of MM cells. Clinically, MM patients are highly susceptible to serious infections due to immunodeficiency [33, 34]. Chloramphenicol may be used to control infections in these patients as well as to suppress their tumors. This anticancer mechanism differs from that of drugs currently used to treat MM and is worthy of further study in the future. Indeed, our findings indicate chloramphenicol may be an effective “new” cytotoxic agent for treatment of myeloma.

## MATRIALS AND METHODS

### Healthy donor samples, patient samples, and human myeloma cell lines

After the study protocol was approved by the institutional review board at Longgang District People's Hospital of Shenzhen, we collected peripheral blood of healthy donors and bone marrow aspirates from nine patients with MM. Healthy and patient volunteers all provided informed consent obtained in accordance with the Declaration of Helsinki. The U266 human myeloma cell line was kindly provided by Dr. Yang Xu (Suzhou University, Jiangsu, China). The RPMI8226 cell line was from the American Type Culture Collection (ATCC, Manassas, VA, USA).

### Reagent

Chloramphenicol was purchased from Sigma (St. Louis, MO) and dissolved in alcohol. The chloramphenicol solutions used in this study were freshly prepared and protected from light. The final concentration of alcohol in the test tubes was less than 0.5% (v/v).

### Proliferation and clonogenic assays of MM cells

To establish the dose- and time-dependent responses to chloramphenicol, MM cells (1 × 10^4^/well) were plated in round-bottom 96-well plates and incubated in 200 μL of medium containing the indicated concentration of chloramphenicol (0, 25, 50, 100, 200, 300, and 400 μg/mL for the dose-response assays; 48 h at 100 μg/mL for the time-response assays). Proliferation of treated and untreated MM cells was assessed using colorimetric assays (Cell Counting Kit-8; Dojindo, Kumamoto, Japan). Briefly, 20 μL of CCK-8 were added to each well and incubated for 3–4 h at 37°C in a humidified CO_2_ incubator. The absorbances at 450 nm and 630 nm were monitored using a microplate reader (ELX800, BIO-TEK, USA).

To determine the capacity of chloramphenicol to inhibit clonogenic tumor cell growth, U266 and RPMI8226 MM cells were plated in quadruplicate in 35 mm^2^ tissue culture dishes (tumor cells: 10,000/well) and incubated in the presence of different chloramphenicol concentrations in RPMI-1640 medium supplemented with 0.9% methylcellulose, 30% fetal bovine serum (FBS), 2 mM of l-glutamine, and 20 μg/mL of gentamycin sulfate. The cells were incubated for 2–3 weeks at 37°C under a 5% CO_2_ atmosphere, after which colonies consisting of > 40 cells were counted under a microscope.

### Trypan blue exclusion cell viability assays

Ten microliters of 4% trypan blue exclusion dye were added to 90 μL of treated or untreated tumor cells and examined under a microscope. Numbers of viable cells were then estimated using a hemocytometer.

### Cellular ATP content measurement

Total cellular ATP levels were determined using an ATP kit assay. MM cell lysates were prepared according to the manufacturer's protocol (BioVision, Mountain View, CA). Each reaction was performed by mixing 5–10 μL of lysate with 90 μL of reaction buffer in a 96-well plate. Finally, luciferase and its substrates were added, and luminescence intensity was immediately measured using a luminometer (TopCount; Packard, Ramsey, MN) calibrated using appropriate ATP standards.

### Analysis of apoptotic cell death

To assess apoptotic cell death, cells were initially washed twice in washing buffer (8 g NaCl, 0.2 g KCl, 1.44 g Na_2_HPO_4_, 0.24 g KH_2_PO4, and 1 L of H_2_O; pH 7.2) and resuspended in 400 μL of Dulbecco's PBS. Thereafter, 100 μL aliquots of the cell suspension were incubated with 10 μL of 50 μg/mL propidium iodide and 5 μL of annexin-V-FITC for 15 min at room temperature in the dark. The cells were then analyzed using flow cytometry.

### Western blotting

For western blotting, MM cells were lysed in sodium dodecyl sulfate (SDS) loading buffer, and aliquots of the resultant lysate were loaded onto 10% SDS polyacrylamide gel. After electrophoresis, the proteins in the gel were electrotransferred to nitrocellulose sheets; probed for 2 h with primary antibodies against cytochrome C (Cytc), cleaved caspase 9, and cleaved caspase 3 (Cell Signaling Technology, Danyers, MA, USA); diluted 1/1000 in PBS-T with 5% milk (Santa Cruz Biotechnology, Santa Cruz, CA) at room temperature; washed three times with PBS-T; incubated with a secondary antibody (HRP-conjugated anti-rabbit IgG) for 1 h at room temperature; washed three times with PBS-T; dried and incubated for 1 min with enhanced chemiluminescence reagent (Santa Cruz Biotechnology); and visualized in a Kodak Imager (KodakFilm, Kodak, USA). The caspase inhibitor z-VAD-FMK was purchased from ICN (USA).

### *In vitro* invasion assays

*In vitro* invasion assays were carried out in transwell plates using membranes with 8 μm pores, as previously described [10]. Briefly, each transwell membrane was pre-coated with 25–30 μL of diluted Matrigel (BD Biosciences, Belford, MA) (1:3 dilution with a serum-free DMEM), after which RPMI8266 or U266 cells (2.5 × 10^4^ cells per well) were seeded on top of the Matrigel layer and incubated for 48 h. Non-invading cells and the Matrigel layer were then removed using a cotton bud, and the migrated cells, attached to the lower side of the membrane, were fixed by immersion in 4% formaldehyde for 3 min. Finally, the invading cells were counterstained with 0.05% crystal violet and counted.

### Proliferation and clonogenic assay of primary tumor cells

Mononuclear cells (MNCs) were isolated from bone marrow samples using density gradient centrifugation. The CD138^+^ and CD138^−^ fractions were then isolated from the MNCs using CD138 microbeads (Miltenyi Biotec) and an AutoMACS magnetic cell sorter (Miltenyi Biotec). The CD138^−^ fraction was further depleted of normal hematopoietic progenitors using CD34, CD3, CD4 and CD8 microbeads (Miltenyi Biotec). The resulting two fractions (CD138^+^CD34^−^CD3^−^CD4^−^CD8^−^ and CD138^−^ CD34^−^CD3^−^CD4^−^CD8^−^ cells; 2.5 × 10^5^/mL) were plated with or without different concentrations of chloramphenicol in round-bottom 96-well plates for colorimetric assays or in a methylcellulose culture system containing rhIL-6 (10 ng/mL, PeproTech) for clonogenic assays (1 × 10^5^ cells per well). Tumor cell colonies were counted after 2–3 weeks of culture. As a control for MM cells, CD34^+^ progenitor cells from one donor's peripheral blood were cultured in a methylcellulose culture system with 50 ng/ml GM-CSF (cells: 100,000/well). The phenotype of the cells in these colonies was confirmed by flow cytometry.

### Statistical analysis

Differences between groups were assessed using Student's *t* test or one-way analysis of variance (ANOVA) with post hoc multiple comparisons test. Values of *P* < 0.05 were considered significant.
